# Intriguing Periprosthetic Fracture of Hip Stem and Proximal Femoral Replacement

**DOI:** 10.7759/cureus.2496

**Published:** 2018-04-17

**Authors:** MN Baig, Abdul Halim Dzufar, Colin G Murphy, Bill Curtin

**Affiliations:** 1 Department of Trauma and Orthopaedic Surgery, Galway University Hospital , Galway, IRL; 2 Department of Trauma and Orthopaedic Surgery, Galway University Hospital, Galway, IRL

**Keywords:** vancouver, megaprosthesis, endoprosthesis, proximal femoral replacement

## Abstract

Lateral femoral prosthesis perforation is an uncommon periprosthetic fracture. Periprosthetic fractures may be fixed with open reduction and internal fixation, or with revision arthroplasty, depending on the type of fracture, the condition of the host bone in the proximal femur, the stability of the implant, and occasionally the medical co-morbidities of the patient. Proximal femoral replacement is a complex and challenging procedure but provides a better chance of early mobilisation. We describe a case of treating a 71-year-old woman who presented with an unusual type of periprosthetic fracture, treated with a revision arthroplasty procedure using a proximal femur replacement.

## Introduction

The incidence of peri-prosthetic fractures is rising due to an increase in the number of arthroplasty procedures for elderly patients. The Vancouver classification is used to describe the location and stability of the fractures [1]. It is based on three main parameters on the basis of the characteristics of a peri-prosthetic fracture. The three main parameters are the stability of the prosthesis, location of the fracture, and quality of surrounding bone. It is a simple, easy, and validated classification system, but there can sometimes be a difference in inter-observer sub classification of type peri-prosthetic fractures. Proximal femoral replacement, similar to what was done in our case, is a segmental modular system for the revision of hip replacements or peri-prosthetic fractures, and it can be a challenging procedure.

## Case presentation

A 71-year-old woman with a history of a right hip intra-capsular fracture in 2006 presented to the emergency department after a fall at her home. In 2006 she had a DHS (dynamic hip screw) as a result of right hip intra-capsular fracture. She developed osteonecrosis which led to a total hip replacement in 2010. Her medical history was relevant for hypertension and had a left mastectomy 25 years ago due to breast cancer. Prior to this episode she denied any trouble with this hip since her surgery in 2010.

**Figure 1:**
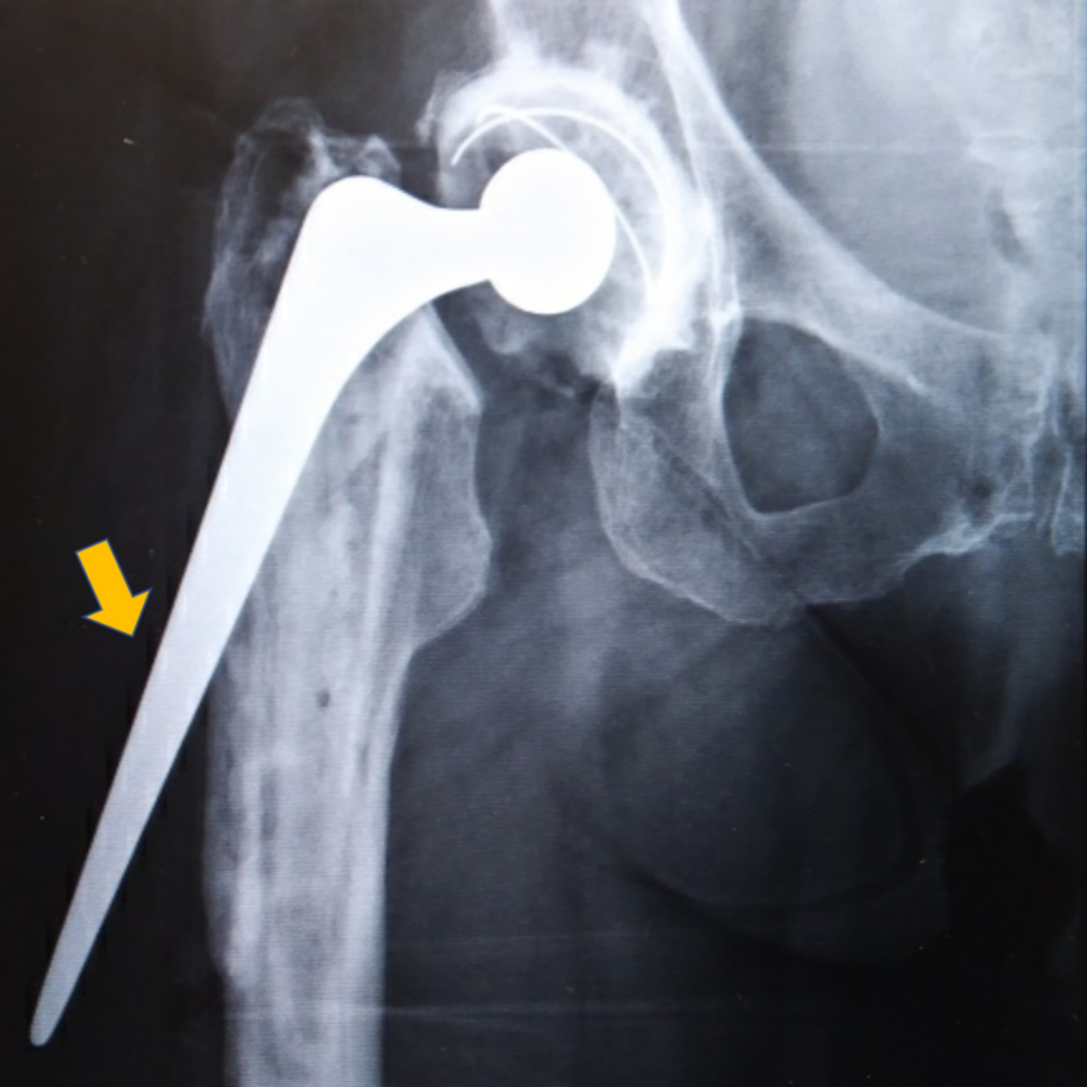
Periprosthetic Fracture Vancouver B3 periprosthetic fracture with protrusion of the femoral implant through the cement and lateral wall of the proximal femur.

**Figure 2:**
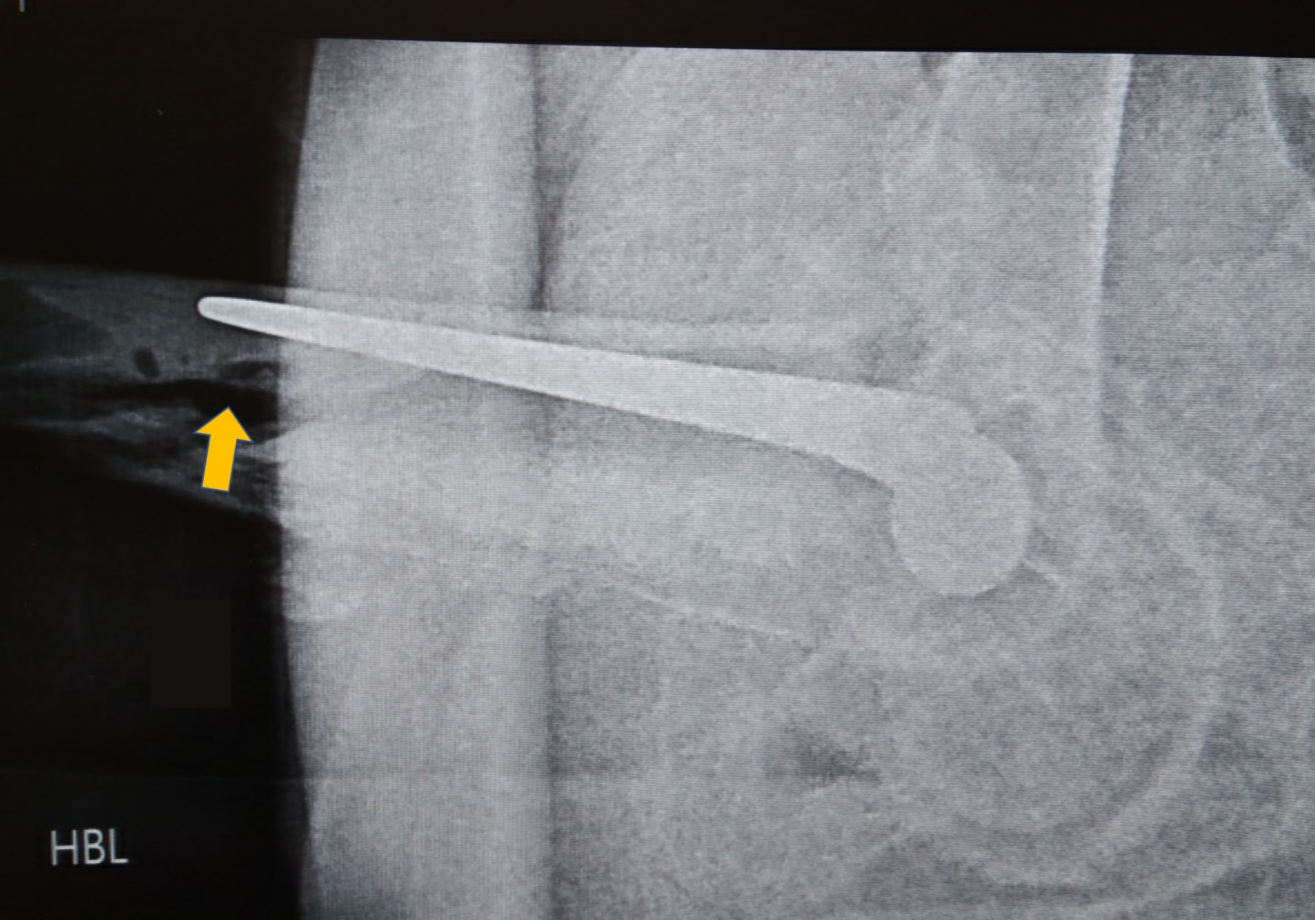
Periprosthetic Fracture, Lateral View Lateral view showing extensive comminution of the proximal femur, and disruption of the bone-cement, and cement-prosthesis interface respectively.

Her radiographs demonstrate a Vancouver B3 peri-prosthetic fracture (Figures 1-2), with lateral extrusion of the highly polished double taper stem through the cement mantle and through lateral wall of the proximal femur. Two treatment options were considered;

i)                    A femoral component revision with an allograft and

ii)                   A proximal femoral replacement.

The former option is often preferred for low-demand patients with extensive medical co-morbidities, while the latter- while representing a larger surgical insult for the patient- facilitates immediate weight bearing and early rehabilitation.

**Figure 3:**
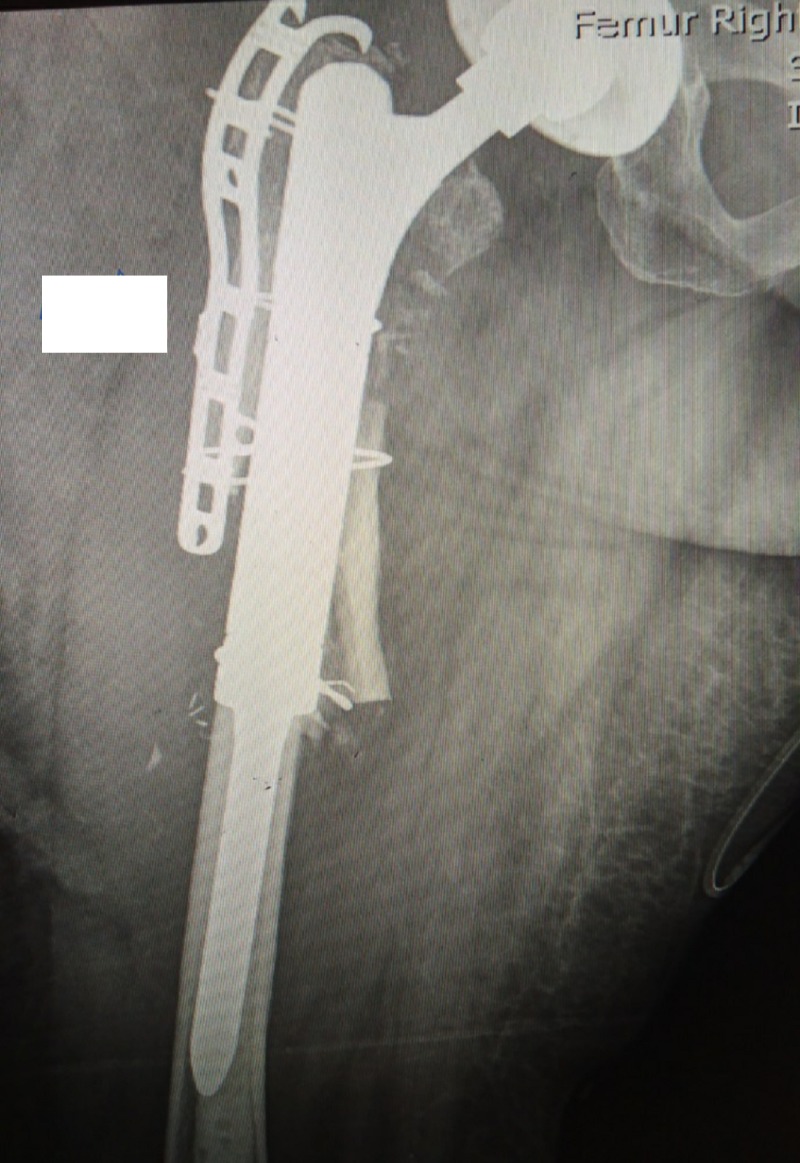
Proximal Femoral Replacement Proximal Femoral Replacement, with trochanteric claw plate and dall miles cables supplementation.

The patient  underwent a both component revision arthroplasty procedure (Figure 3); using an the multiple fracture lines already present through the proximal femur in lieu of an extended trochanteric osteotomy, the prosthesis and cement were removed from the proximal femur, and a modular endoprosthesis (LPS ® DePuy Limb Preservation System (Warsaw, IN, USA) proximal femoral replacement) inserted. The acetabular component was also revised. While representing an addition extra step and a slightly increased magnitude of the surgical insult, it allows use of a larger head, and the optimsation of any version issues to reduce the risk of post-operative dislocation. An additional trochanteric claw plate was used to re-attach the bone of the proximal femur to the prosthesis, thus ensuring good abductor function. The patient tolerated the surgery without incident. She was able to commence immediate full weight-bearing, protected with a Zimmer frame, on postoperative day one. At her six-week postoperative evaluation, she was ambulating independently, though had continued with the use of her walking frame for ‘balance and confidence’.

## Discussion

The use of proximal femoral replacement prostheses for peri-prosthetic fracture is becoming more common with the rising incidence of peri-propsthetic fractures, in particular for those patients with poor femoral bone stock, but there are important factors to consider before choosing this procedure [2-3]. Most importantly, a discussion should take place between the surgeon and the patient to establish appropriate expectations and requirements. Patients should be thoroughly examined, including noting previous scars, the status of abductors, and limb length. Preoperative templating helps in estimating prosthetic requirements. Surgeons should be careful to minimise soft tissue dissection off the native bone and retain as much of the host bone as possible. Restoring appropriate leg length and soft tissue tension are challenging.

Proximal femoral replacement is a form of device generically known as megaprosthesis or endoprosthesis [3-4]. The modern version of these devices is highly modular, and  can be customized according to the patient's native anatomy, with multiple options for offset, neck length and version.. The main indications for use of this are fractures and non-unions with massive bone loss or comminution, bone tumours, metastatic bone disease, or failed arthroplasty, as in this case discussed above.

## Conclusions

This case discusses a complex and uncommon peri-prosthetic fracture. Proximal femoral replacement, with a cemented stemmed diaphyseal-bearing modular endoprostheis while technically challenging, represents a useful solution to the particular challenge of this particular peri-prosthetic fracture configuration.

## References

[1] Fleischman AN, Chen AF (2015). Periprosthetic fractures around the femoral stem: overcoming challenges and avoiding pitfalls. Ann Transl Med.

[2] Curtin M, Bryan C, Murphy E (2016). Early results of the LPS™ limb preservation system in the management of periprosthetic femoral fractures. J Orthop.

[3] Baig MN, Curtin W, Callaghan MA, Murphy CG (2017). Catastrophic cement reaction following cementation for megaprosthesis for proximal femoral fracture. BMJ Case Rep.

[4] Parvizi J, Sim FH (2004). Proximal femoral replacements with megaprostheses. Clin Orthop Relat Res.

